# Validation and Classification of Atypical Splicing Variants Associated With Osteogenesis Imperfecta

**DOI:** 10.3389/fgene.2019.00979

**Published:** 2019-10-18

**Authors:** Lulu Li, Yixuan Cao, Feiyue Zhao, Bin Mao, Xiuzhi Ren, Yanzhou Wang, Yun Guan, Yi You, Shan Li, Tao Yang, Xiuli Zhao

**Affiliations:** ^1^Department of Medical Genetics, Institute of Basic Medical Sciences, Chinese Academy of Medical Sciences & School of Basic Medicine, Peking Union Medical College, Beijing, China; ^2^Department of Orthopaedics, The People’s Hospital of Wuqing District, Tianjin, China; ^3^Department of Pediatric Orthopaedics, Shandong Provincial Hospital Affiliated to Shandong University, Jinan, China; ^4^Department of Anesthesiology and Critical Care Medicine, Johns Hopkins University, School of Medicine, Baltimore, MD, United States

**Keywords:** osteogenesis imperfecta, *COL1A1*, *COL1A2*, minigene splicing assay, atypical splicing variants

## Abstract

Osteogenesis Imperfecta (OI) is a rare inherited bone dysplasia, which is mainly caused by mutations in genes encoding type I collagen including *COL1A1* and *COL1A2*. It has been well established to identify the classical variants as well as consensus splicing-site-variants in these genes in our previous studies. However, how atypical variants affect splicing in OI patients remains unclear. From a cohort of 867 OI patients, we collected blood samples from 34 probands which contain 29 variants that are located close to splice donor/acceptor sites in either *COL1A1* or *COL1A2*. By conducting minigene assay and sequencing analysis, we found that 17 out of 29 variants led to aberrant splicing effects, while no remarkable aberrant splicing effect was observed in the remaining 12 variants. Among the 17 variants that affect splicing, 14 variants led to single splicing influence: 9 led to exon skipping, 2 resulted in truncated exon, and 3 caused intron retention. There were three complicated cases showing more than one mutant transcript caused by recognition of several different splice sites. This functional study expands our knowledge of atypical splicing variants, and emphasizes the importance of clarifying the splicing effect for variants near exon/intron boundaries in OI.

## Introduction

Osteogenesis imperfecta (OI), also known as brittle bone disease, is an inherited skeletal dysplasia characterized by frequent fractures, blue sclerae, bone deformity, and relaxation of skin and ligament. OI is considered as a rare bone disease and its prevalence is reported to be 1 in 15,000 live births (Stoll et al., 1989). Based on phenotypes, patients with OI can be categorized into 4 types according to Sillence et al. (1979): patients with the mildest phenotype and with blue sclerae (type I); lethal (type II); the severe form with progressively skeletal deformity (type III); moderate OI with variable bone deformity (type IV). Recently types V–XIX OI were grouped according to genetic and clinical characteristics (Rauch and Glorieux, 2004; Forlino and Marini, 2016; Lindert et al., 2016; Marini et al., 2017).

OI is mainly caused by abnormal structure and quantity of type I collagen, functioning as the main matrix in bone tissue. Type I collagen is encoded by *COL1A1* (MIM# 120150) and *COL1A2* (MIM# 120160) (Marini et al., 2007). Mutations in other collagen-related genes have been reported to contribute to OI development as well, including *IFITM5*, *SERPINF1*, *CRTAP*, *P3H1*, *PPIB*, *SERPINH1*, *FKBP10*, *PLOD2*, *BMP1*, *SP7*, *TMEM38B*, *WNT1*, *CREB3L1*, *SPARC*, and *MBTPS2* (Byers and Pyott, 2012; Rohrbach and Giunta, 2012; Lindert et al., 2016; Gagliardi et al., 2017). Nevertheless, around 90% of OI are autosomal dominant inheritance with a familial history and are caused by mutations in *COL1A1* and *COL1A2*.

Typical mutation spectrum of OI includes missense, nonsense, frameshift, and splice site mutations. Despite of these classical mutations, it was shown that a large portion of DNA variants disrupted splicing in cancer-related diseases (Sanz et al., 2010). However, it was rarely reported whether similar DNA variants have an impact on aberrant splicing in OI patients. RNA splicing is essential for transcription processing and for the correct protein synthesis. Human genes undergo alternative splicing therefore different transcripts can be generated (Johnson et al., 2003). The process of splicing initiates from recognition of core splicing signal, including splicing donor (gt), splicing acceptor (ag) and a branch point (Wang and Burge, 2008). The splicing process is catalyzed by the spliceosome, which contains five uridine rich ribonucleoproteins (U1, U2, U4, U5, and U6) and more than 200 associated proteins (Zhou et al., 2002). During the splicing process, a cryptic splice site may be activated due to the variants and generate aberrant splicing products (Sun and Chasin, 2000). Therefore, studying the splicing effects caused by the variants is important for understanding the pathogenesis and molecular mechanisms of OI.

Because of the very low expression levels of *COL1A1/COL1A2* in peripheral blood, RNAs from the tissue of OI patients would be ideal for examining whether the variants can affect RNA splicing. However, the availability of the tissue of OI patients is limited. Therefore, a minigene assay, which is based on patients’ genomic DNA, represents a valid and powerful approach to study the splicing pattern (Cooper, 2005; Ahlborn et al., 2015; Fraile-Bethencourt et al., 2019).

It has been reported that variants at splicing sites can drive to splicing effects in some OI patients (Schleit et al., 2015; Schwarze et al., 1999). However, most of these variants were typical splicing variants which were located at splicing donor/acceptor sites in introns. A recent study reported splicing effects in 40 OI patients harboring the variants in introns (Schleit et al., 2015). Although the pathogenicity of variants at splicing sites has been well studied, atypical splicing sites beyond the splicing sites (GT-AG) were rarely reported. To determine whether a variant has an impact on splicing efficiency, we selected 34 OI probands carrying 29 different variants which were located close to the splicing sites in introns or exons of *COL1A1* or *COL1A2*. Based on minigene assays and sequence analysis, 17 variants showed aberrant splicing effects while 12 variants presented no splicing consequences. The aberrant splicing was further classified into 3 patterns: exon skipping, truncated exon/intron retention resulted from recognition of alternative splice sites and compound aberrant splicing. Current findings enriched the splicing patterns, and suggested that atypical splicing variants may represent a large group of pathogenic mutations of OI. 

## Methods and Materials

### Variant Nomenclature

The variants of *COL1A1* and *COL1A2* were named according to variant nomenclature provided by Human Genome Variation Society (http://www.hgvs.org/munomen). The genomic DNA and cDNA sequences of *COL1A1* (NC_000017.11) and *COL1A2* (NC_000007.14) were obtained from National Center for Biotechnology Information (NCBI) reference sequence and University of California, Santa Cruz (UCSC) Genome browser database (http://genome.ucsc.edu/). The altered proteins were named based on the sequencing of mutant transcripts.

### Subjects

A total number of 867 patients (from 489 families) diagnosed as OI were recruited for this study from 2014 to 2018. Information of their phenotypes, including number of fractures, blue sclerae, affected skeletal location, and bone deformity were recorded after obtaining patients’ informed consent. Tissue samples, including peripheral blood and/or skin, were collected to detect the variants. After sequence analysis, 34 probands from different families carrying *COL1A1* or *COL1A2* variants close to the exon/intron boundaries were enrolled for minigene splicing assay. All variants identified in this study have been submitted to the Osteogenesis Imperfecta Variant Database (http://oi.gene.le.ac.uk/). 

### *In Silico* Analysis

Online software ESE Finder 3.0 and Human Splicing Finder (version 3.1) were used to predict the splicing effect of each of the variants. Analysis of ESE Finder was performed to detect exonic splicing enhancers for SR proteins as well as alterations in splice sites. SRProteins matrix library was used to analyze the variants located in exons and SpliceSites matrix library was used for variants in introns. All analyses were performed with default threshold values.

### Whole Exome Sequencing (WES)

Genomic DNA was extracted from the peripheral blood, and 1–3 μg genomic DNA was used for WES as described previously (Li et al., 2019). Sequencing was carried out on HiSeq 4000 System (Illumina) as 150 bp paired-end runs after DNA fragmentation, end pair ligation, purification and size distribution assessment. Sequencing analysis was performed using the Pipeline (version 1.3.4; Illumina).

### Sanger Sequencing

Sanger sequencing was employed to verify the variants in *COL1A1* and *COL1A2* after WES, and to verify the splicing variants after minigene assay. The process was described previously (You et al., 2018). Briefly, genomic DNA was isolated using a proteinase K and phenol–chloroform method. Primers were designed by Primer3 (http://primer3.ut.ee/). Sequencing was conducted in Applied Biosystems 3730xl DNA Analyzer (Thermo Fisher Scientific, Waltham, MA, USA). Result of Sanger sequencing was analyzed using CodonCode Aligner (version 6.0.2.6; CodonCode, Centerville, MA, USA). The sequence results were aligned to reference sequences *COL1A1* (NC_000017.11) and *COL1A2* (NC_000007.14) and DNA alignment was conducted using DNAman (version 6.0, LynnonBiosoft, USA).

### Minigene Assay

**Figure 1:**
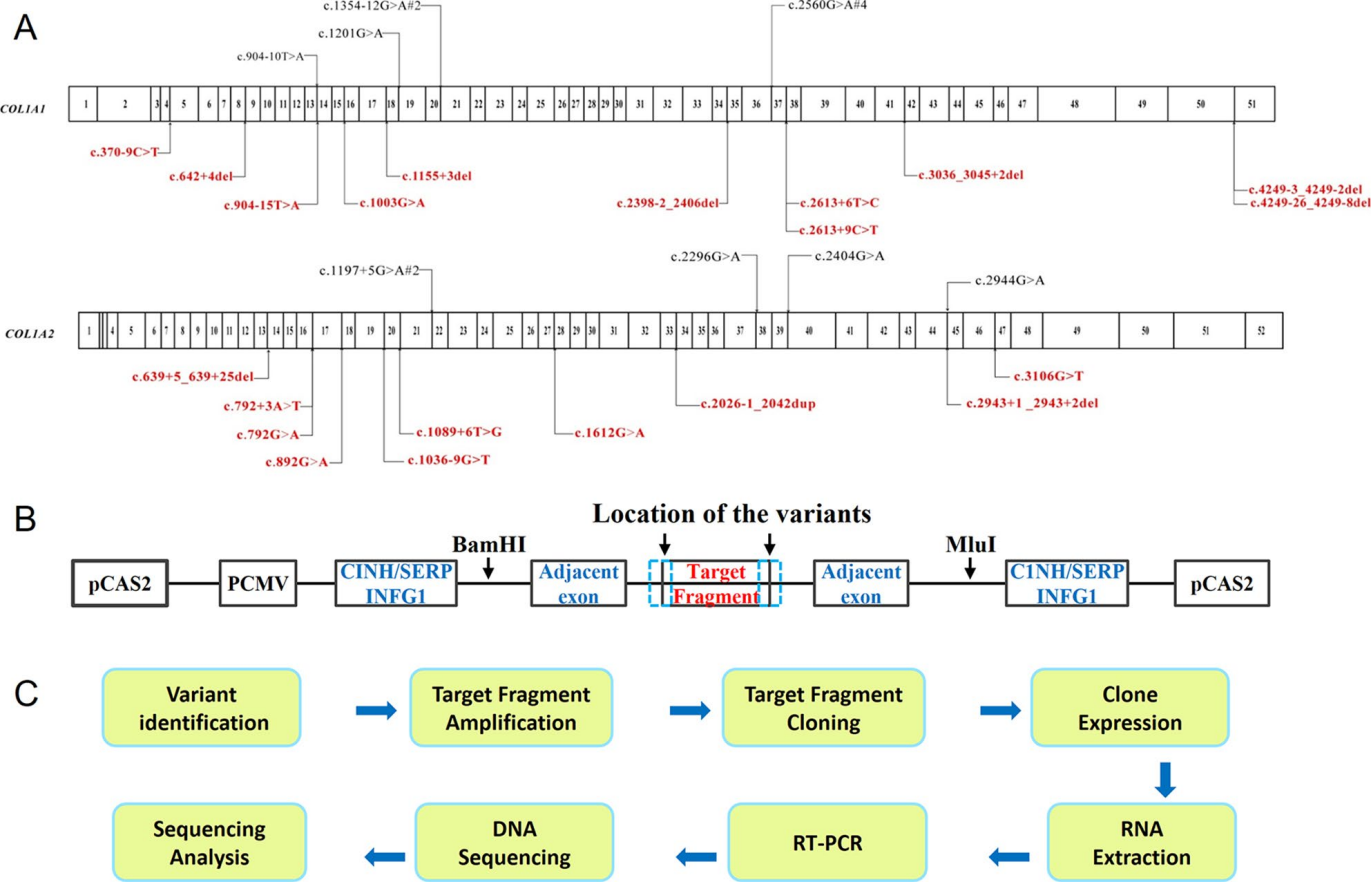
Schematic map of the procedure of minigene assay. **(A)** The distribution of mutations identified in *COL1A1* and *COL1A2* in OI patients. The variants at exon–intron boundary were labeled on the map, including both the annotated variants (shown at the top in black) which can be found in HGMD, as well as novel variants (shown at the bottom in red). The numbers in the cartoon represent the exons within *COL1A1* or *COL1A2*. **(B)** Overview of pCAS2 (Upper panel) vector constructs. **(C)** Experimental procedure of minigene assay.

Twenty-nine variants close to intron–exon boundary in *COL1A1* and *COL1A2* from 34 probands were selected for the minigene splicing assay (**Figure 1A**). The fragments of interests varying from 808 bp to 2,510 bp (**Table S1**) which contain the putative splicing variant along with flanking exons were amplified by high fidelity PCR. The PCR was carried out using HS DNA polymerase (TaKaRa, Shiga, Japan) and forward and reverse primers with restriction sites for BamHI or MluI (New England Biolabs, Ipswich, MA, USA). Primers were designed for each target fragment using Primer3 (http://primer3.ut.ee/) (**Table S1**). The amplified target fragments were cloned into the pCAS2 vector (**Figure 1B**) using restriction endonucleases BamHI, MluI, and T4 DNA ligase (New England Biolabs). The constructed vector was further transformed into *E. coli* DH5α Competent Cells (TaKaRa, Shiga, Japan), followed by sequencing verification. Both the purified constructs of wild type and mutant type were transferred into HEK293T cells using Invitrogen Lipofectamine 3000 Transfection Kit (Thermo Fisher Scientific). HEK293T cell line was selected to eliminate endogenous interference for its low expression of type I collagen. After 24 h incubation, RNA was isolated using Trizol reagent (Invitrogen). One microgram total RNA was used for RT-PCR using PrimeScript RT reagent kit with gDNA Eraser (TaKaRa). PCR products were separated on 1% agarose gel containing ethidium bromide. The target DNA bands were purified using GeneJET Gel Extraction Kit (Thermoscientific, Lithuania), followed by DNA sequencing with ABI3730xl (Thermo Fisher Scientific, Waltham, MA, USA). The procedure was summarized in the schematic map (**Figure 1C**).

### Fibroblasts Assay

Skin samples were collected from probands PUMC-253, 371, 98, 401, and 216 following the skin biopsy process or surgical operation. Cleaned dermal tissues were cut into small pieces of 1 mm^2^ and washed with PBS. After transferring the dermal pieces into a cell culture flask, skin tissue was attached on the flask in humid environment overnight and fibroblasts were cultured in fibroblast culture medium [F12 (Gibco, NY, USA) containing 15% FCS (Gibco, Australia) and 1% antibiotics (Sigma)]. RNA was isolated using Trizol reagent (Invitrogen) when dermal fibroblasts were cultured for 3 passages. After reverse transcription, PCR products were separated on 1% agarose gel followed by sequencing confirmation.

## Results

We enrolled a cohort of 867 OI patients and 72 OI patients (from 26 families) carried 22 different classical splicing mutations (with gt/ag mutations) in *COL1A1* and *COL1A2* (**Table S2**). This research focused on the atypical splicing variants that are located close to intron–exon boundaries, in order to determine whether such variants affect splicing. Details of variants found by whole exome sequencing or Sanger sequencing, expected variant type, actual variant type by minigene analysis, alteration of nucleotide, amino acid change, and the classification of the OI type were shown in **Table 1**. All 34 probands were germline heterozygotes with variation of *COL1A1* or *COL1A2*, and each cell contained a normal allele and a mutant allele. Minigene assay showed that the normal alleles only formed wild type transcripts. So in the following results the transcripts from the mutant alleles will be mainly clarified.

**Table 1:** Splicing analysis of the atypical *COL1A1* variants and atypical *COL1A2* variants.

Proband number	Gene	Location	Mutation	Expect effect	Result of Minigene assay				
					Effect observed in Minigene assay	Nucleotide change	Predicted amino acid change	Fibroblasts assay	OI type
**Variants in introns**									
PUMC-229	*COL1A1*	Intron 4	c.370-9C > T	Splicing	No aberration	No aberration	No aberration	NA	III
PUMC-401	*COL1A1*	Intron 8	c.642+4delA	Splicing	Skipping of exon 8/Skipping of exon 8 and partial exon 9/Retention of intron 7 and skipping of exon 8/26 bp del in exon 8	r.589_642del/r.589_661del/r.587_588ins588+1_588+96/r.617-642del	(p.Gly197_Ser214del)/(p.Gly197Valfs*44)/(p.Pro196_Gly197ins32)/(p.Glu207Serfs*17)	Skipping of exon 8/Retention of intron 7 and skipping of exon 8/26 bp del in exon 8/36 bp del in exon 8/Retention of intron 8	IV
PUMC-15	*COL1A1*	Intron 13	c.904-10T > A	Splicing	Retention of 8bp	r.369_370ins370-8_370-1	(p.Gly302Valfs*242)	NA	I
PUMC-105	*COL1A1*	Intron 13	c.904-15T > A	Splicing	Retention of 13bp	r.903_904ins904-13_904-1	(p.Gly302Valfs*12)	NA	I
PUMC-186	*COL1A1*	Intron 17	c.1155+3delA	Splicing	Skipping of exon 17	r.1057_1155del	(p.Gly353_Ala385del)	NA	I
PUMC-369	*COL1A1*	Intron 20	c.1354-12G > A	Splicing	Retention of 10bp	r.903_904ins904-10_904-1	(p.Gly452Profs*26)	NA	I
PUMC-189	*COL1A1*	Intron 20	c.1354-12G > A	Splicing	Retention of 10bp	r.903_904ins904-10_904-1	(p.Gly452Profs*26)	NA	I
PUMC-111	*COL1A1*	Intron 34-exon 35	c.2398-2_2406del	Splicing	Skipping of exon 35	r.2398_2451del	(p.Gly800_Pro817del)	NA	IV
PUMC-109	*COL1A1*	Intron 37	c.2613+6T > C	Splicing	Skipping of exon 37	r.2560_2613del	(p.Gly854_Pro871del)	NA	IV
PUMC-469	*COL1A1*	Intron 37	c.2613+9C > T	Splicing	No aberration	No aberration	No aberration	NA	III
PUMC-480	*COL1A1*	Exon 41-intron 41	c.3036_3045+2del	Splicing	Skipping of exon 41/partial exon 41 del	r.2938_3045del/r.3029_3045del	(p.Gly980_Glu1015del)/(p.Glu1011Glyfs*19)	NA	IV
PUMC-276	*COL1A1*	Intron 50	c.4249-26_4249-8del	Splicing	Exon 51_3′UTRdel	r.4249_4395+1148del	(p.Thr1417Ala*64)	NA	IV
PUMC-290	*COL1A1*	Intron 50	c.4249-3_4249-2del	Splicing	Exon 51_3′UTRdel	r.4249_4395+1148del	(p.Thr1417Ala*64)	NA	IV
PUMC-391	*COL1A2*	Intron 13	c.639+5_639+25del	Splicing	Skipping of exon 13	r.595_639del	(p.Gly199_Thr213del)	NA	I
PUMC-90	*COL1A2*	Intron 16	c.792+3A > T	Splicing	Skipping of exon 16	r.739_792del	(p.Gly247_Lys264del)	NA	III
PUMC-224	*COL1A2*	Intron 19	c.1036 -9G > T	Splicing	No aberration	No aberration	No aberration	NA	IV
PUMC-98	*COL1A2*	Intron 20	c.1089+6T > G	Splicing	Skipping of exon 20	r.1036_1089del	(p.Gly346_Pro363del)	Skipping of exon 20	I
PUMC-216	*COL1A2*	Intron 21	c.1197+5G > A	Splicing	Skipping of exon 21	r.1090_1197del	(p.Gly364_Arg399del)	Skipping of exon 21	IV
PUMC-37	*COL1A2*	Intron 21	c.1197+5G > A	Splicing	Skipping of exon 21	r.1090_1197del	(p.Gly364_Arg399del)	NA	IV
PUMC-253	*COL1A2*	Intron 33–exon 34	c.2026-1_2042dup	Splicing	No aberration	r.2042_2043insertGGGTGCTCCTGGTGCTGT	(p.Val681_Gly682insGlyAlaProGlyAlaVal)	No aberration	IV
PUMC-312	*COL1A2*	Intron 44	c.2943+1-2943+2delgt	Splicing	Skipping of exon 44	r.2836_2943del	(p. Gly946_Thr981del)	NA	III
**Variants in exons**									
PUMC-234	*COL1A1*	Exon 16	c.1003G > A	Missense	No aberration	c.1003G > A	No aberration	NA	IV
PUMC-2	*COL1A1*	Exon 19	c.1201G > A	Missense	No aberration	c.1201G > A	No aberration	NA	III
PUMC-351	*COL1A1*	Exon 37	c.2560G > A	Missense	No aberration	c.2560G > A	No aberration	NA	IV
PUMC-23	*COL1A1*	Exon 37	c.2560G > A	Missense	No aberration	c.2560G > A	No aberration	NA	IV
PUMC-41	*COL1A1*	Exon 37	c.2560G > A	Missense	No aberration	c.2560G > A	No aberration	NA	IV
PUMC-339	*COL1A1*	Exon 37	c.2560G > A	Missense	No aberration	c.2560G > A	No aberration	NA	IV
PUMC-371	*COL1A2*	Exon 16	c.792G > A	Synonymous	Skipping of exon 16	c.739_792del	(p.247Gly_264Lysdel)	Skipping of exon 16	III
PUMC-479	*COL1A2*	Exon 18	c.892G > A	Missense	No aberration	c.892G > A	No aberration	NA	I
PUMC-448	*COL1A2*	Exon 28	c.1612G > A	Missense	No aberration	c.1612G > A	No aberration	NA	III
PUMC-441	*COL1A2*	Exon 38	c.2296G > A	Missense	No aberration	c.2296G > A	No aberration	NA	IV
PUMC-296	*COL1A2*	Exon 40	c.2404G > A	Missense	Retention of 49bp/No aberration	c.2403_2404ins2404-49_2404-1/c.2404G > A	(p.Gly802Alafs*5)/No aberration	NA	I
PUMC-430	*COL1A2*	Exon 45	c.2944G > A	Missense	No aberration	c.2944G > A	No aberration	NA	III
PUMC-115	*COL1A2*	Exon 48	c.3106G > T	Missense	No aberration	c.3106G > T	No aberration	NA	IV

## Splicing Effect Analyzed by Minigene Assay

**Figure 2:**
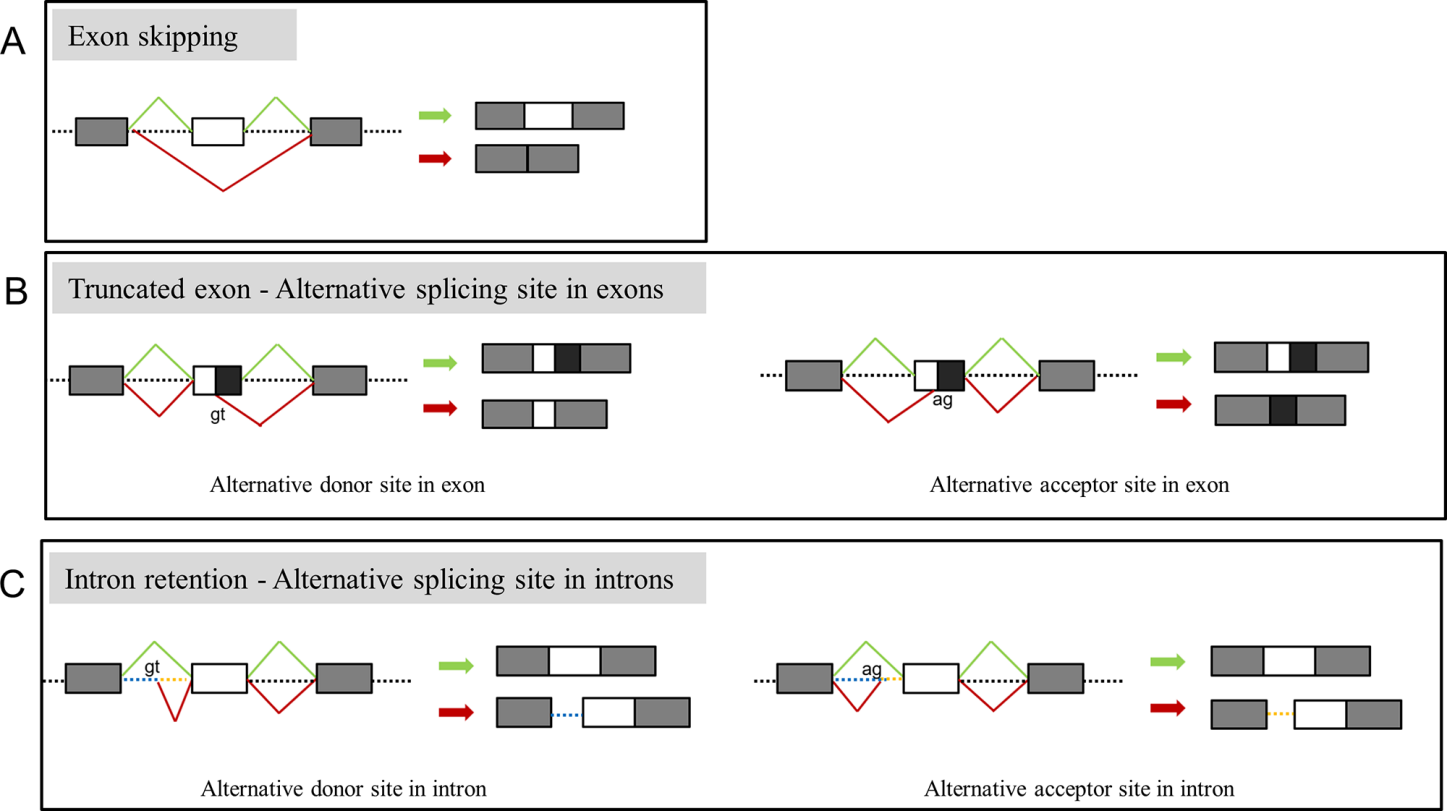
Representation of main splicing effects in OI patients. **(A)** Variants resulting in exon skipping. **(B)** Variants resulting in truncated exon caused by recognition of alternative donor (left) or acceptor (right) in exons. **(C)** Variants resulting in intron retention caused by recognition of alternative donor (left) or acceptor (right) in introns. Splicing products in green indicate the wild type transcript, products in red indicate the aberrant splicing.

Among the 34 probands, there were 29 different variants and 17 variants displayed aberrant splicing based on findings in minigene assay and 12 did not show any splicing consequence (**Table 1**). RT-PCR of RNA extracted from fibroblasts was also conducted for 5 variants (c.642+4delA in *COL1A1*, c.1089+6T > G in *COL1A2*, c.1197+5G > A in *COL1A2*, c.2026-1_2042dup in *COL1A2*, c.792G > A in *COL1A2*) (**Table 1**), and results from fibroblasts were in line with findings of minigene assay. In general, two main types of single-splicing-effects were categorized: exon skipping (**Figure 2A**), and alternative splice sites activation (**Figures 2B, C**). The latter one can be further separated into two subtypes: partial exon deletion resulted from the alternative splice sites in exons (**Figure 2B**), and intron retention caused by alternative splice sites in introns (**Figure 2C**). The results from minigene assay were then compared with the predictions made by *in silico* tools: Human Splicing Finder (version 3.1) and ESE Finder 3.0 (**Table S3**). Both tools only correctly predicted a portion of aberrant splicing, and hence a minigene assay is a solid method to verify the splicing pattern.

### Variants Only Led to Exon Skipping in OI Patients

Nine variants in this study were observed with only exon skipping, as indicated by minigene assay (**Table 1**). These variants include c.1155+3delA, c.2398-2_2406del, c.2613+6T>C in *COL1A1*, and c.639+5_639+25del c.792+3A>T, c.1089+6T>G, c.1197+5G>A, c.2943+1_2943+2delgt, c.792G>A in *COL1A2*. None of these variants drove to frameshift alterations or premature stop codons.

**Figure 3:**
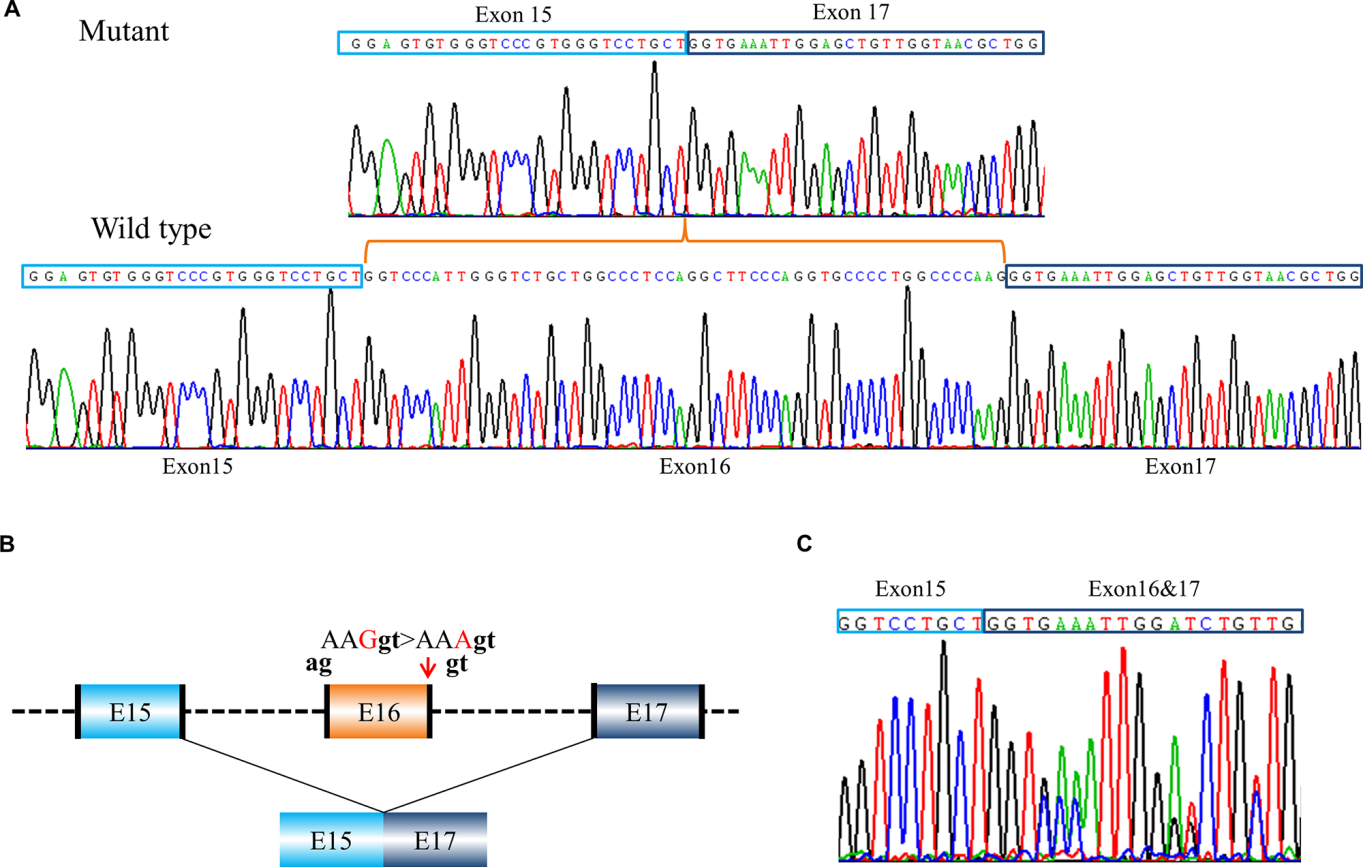
A case of exon skipping resulted from a synonymous mutation (PUMC-371). **(A)** Sequencing analysis by minigene assay indicated a wild type transcript and a mutant transcript. Compared with the wild type transcript, the mutant transcript showed exon 16 skipping. **(B)** Schematic representation of the splicing effect. A synonymous mutation c.792G > A (p.Lys264Lys) in *COL1A2* at the last nucleotide in exon 16 was found by DNA Sanger sequencing. Splicing assay indicated the skipping of exon 16, c.739_792del (p.Gly247_Lys264del). The dinucleotide in black indicated intrinsic splicing donor or acceptor. **(C)** Sequencing analysis of RT-PCR products from patient’s fibroblasts confirmed exon 16 skipping.

Eight of these variants with exon skipping effects are located in introns. Notably, the variant, c.792G > A in *COL1A2* (PUMC-371) in the exon 16 displayed the exon skipping effect as well (**Figure 3**). Generally c.792G > A (p.Lys264Lys) was regarded as a synonymous mutation, but this variant was found at the last nucleotide in exon 16 of *COL1A2*, so we suspect it may affect splicing. Minigene analysis confirmed our conjecture and showed a wild type (**Figure 3A** lower panel) and a mutant transcript (**Figure 3A** upper panel) with exon 16 skipping. The schematic splicing map was shown in **Figure 3B**. To validate the results obtained from the minigene assay, RNA was isolated from skin fibroblasts of the patient, followed by sequencing of RT-PCR products (**Figure 3C**). The endogenous expression was in agreement with findings from minigene assay.

### Partial Exon Deletion Caused by Cryptic Splice Site Activation in Exon

#### Recognition of Alternative Donor Site in Exon

**Figure 4:**
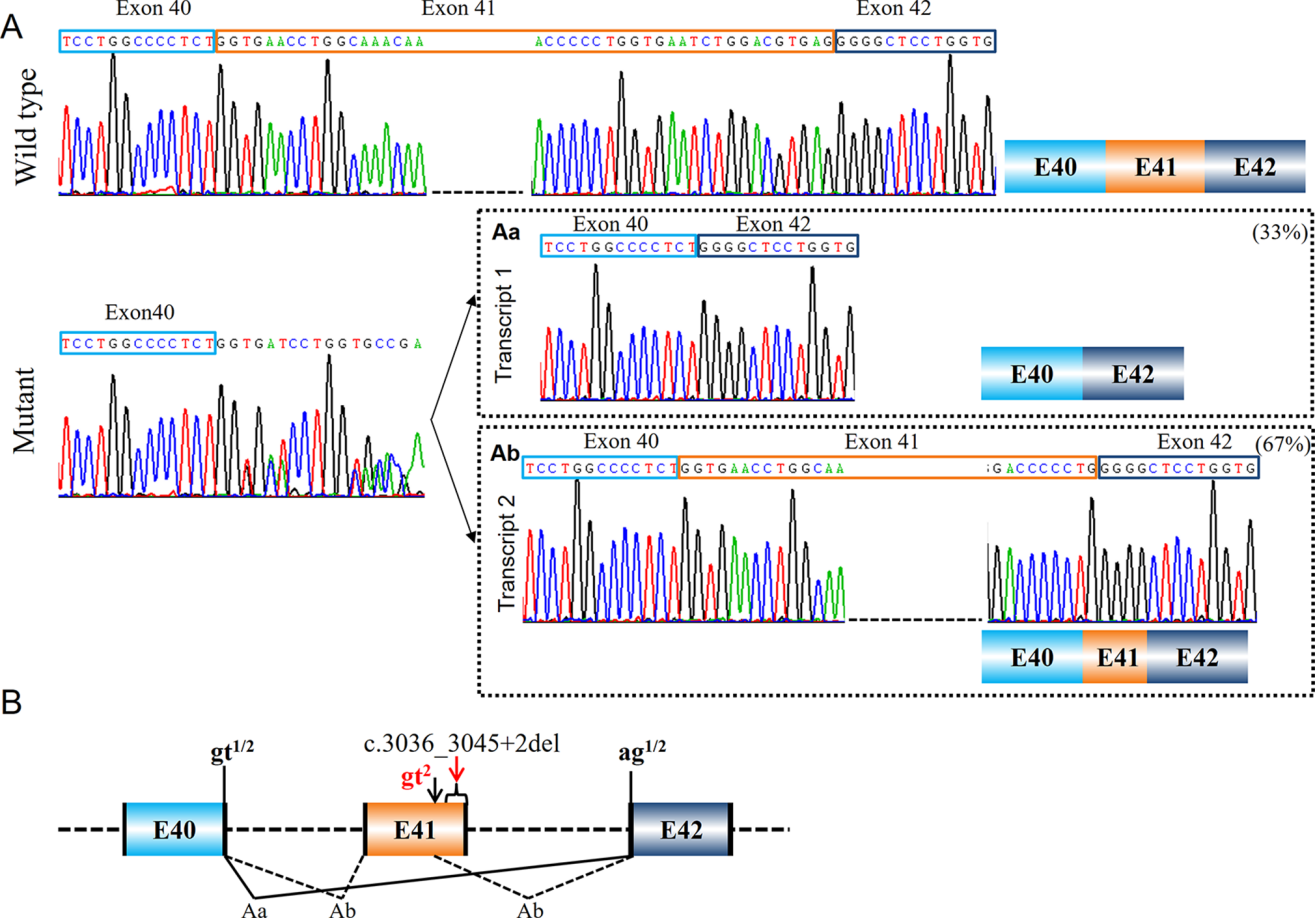
Compound splicing effect with exon skipping and truncated exon (PUMC-480). **(A)** A wild type transcript and a mutant transcript with unspecific signal after exon 40 were detected by minigene analysis. Followed by T clone sequencing, two mutant transcripts were distinguished from the mutant fragments: transcript 1 with skipping of exon 41 **(Aa)**, and transcript 2 with truncated exon 41 caused by recognition of alternative donor site at exon 41 **(Ab)**. **(B)** Schematic representation of splicing effect in this case. Variant c.3036_3045+2del located in exon 41-intron 41 in *COL1A1* was found by DNA Sanger sequencing. Minigene assay showed two different mutant transcripts caused by utilizing alternative splicing donor/acceptor sites. The intrinsic splicing donor gt and acceptor ag were labeled in black and the activated splice sites were labeled in red; all the splice sites used in each mutant transcript was labeled accordingly: gt^1^ indicates the splicing donor site used in transcript 1; gt^2^ indicates the splicing donor site used in transcript 2; ag^1/2^ indicates the splicing acceptor site used in both transcript 1 and 2.

**Figure 5:**
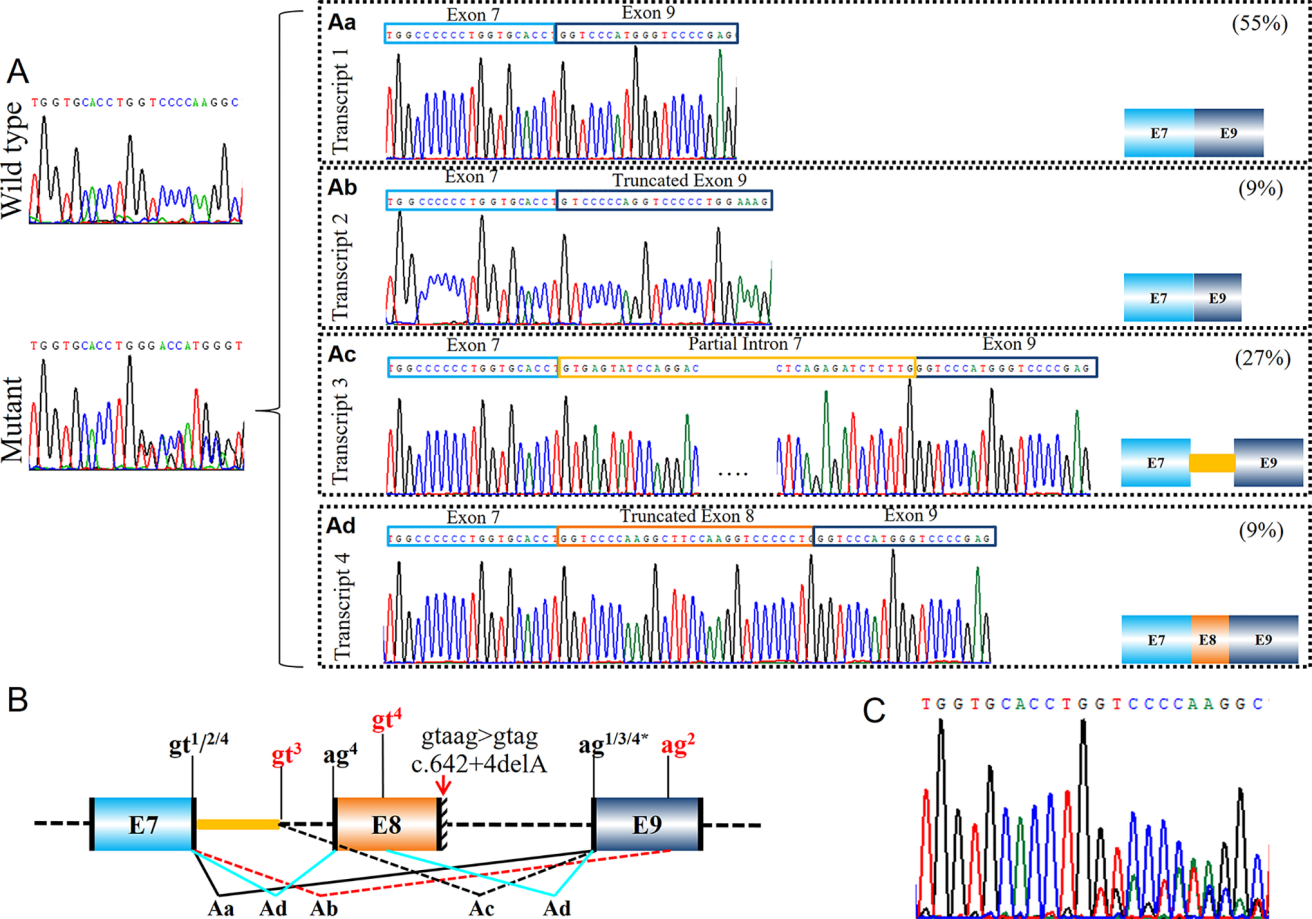
Identification of a complex splicing effect with exon skipping, truncated exon and intron retention (PUMC-401). **(A)** Minigene results indicated a wild type transcript and a mutant transcript. Four different mutant transcripts were further identified by T clone sequencing **(Aa**-**Ad)**. **(B)** Schematic representation of the aberrant splicing effects. Variant c.642+4delA in *COL1A1* was found by DNA Sanger sequencing. Minigene assay indicated four mutant transcripts generated by using different splicing donor/acceptor sites. The intrinsic splicing donor gt and splicing acceptor ag were labeled in black. Both canonical splice sites and cryptic splice sites were marked on the representation: the canonical splice sites in black, and the newly activated splice sites in red. Notations gt^n^ indicates the splicing donor sites utilized in transcripts n; ag^n^ indicates the splicing acceptor sites utilized in transcripts n (n=1-4). (C) Sequencing analysis of RT-PCR products from patient’s fibroblasts confirmed the generation of multiple mutant transcripts.

Variant c.3036_3045+2del in *COL1A1* (PUMC-480) led to the activation of cryptic donor site in the exon (**Figure 4**). Two different transcripts were found by minigene analysis: a wild type transcript from the normal allele and a mutant transcript with disrupted signal after exon 40 (**Figure 4A**). After further T clone sequencing, the mutant transcripts were divided into two segments: only exon 41 skipping in transcript 1 (33%), and a partial skipping of exon 41 in transcript 2 (67%). An alternative donor splice site in exon 41 c.3029_3030 GT was recognized, which led to a truncated exon 41 (**Figure 4Ab**). Variant c.642+4delA in *COL1A1* (PUMC-401) also resulted in the utilization of an alternative donor site (c.617_618GT) and generated truncated exon 8 (**Figure 5Ad**).

#### Recognition of Alternative Acceptor Site in Exon

Three variants were found with alternative splicing acceptor site-induced aberrant splicing. Variant c.642+4delA in *COL1A1* (PUMC-401) was observed that an AG site (c.660_661AG) in exon 9 in *COL1A1* was utilized as the splicing acceptor (**Figures 5Ab, B**). Consequently, a truncated exon 9 was generated. There were two variants c.4249-26_4249-8del in *COL1A1* (PUMC-276) and c.4249-3_4249-2del in *COL1A1* (PUMC-290) which showed the same splicing effects (**Figure S1**). The minigene results of both variants showed an alternative AG site (c.4395+1147_4395+1148AG) in the UTR sequence, which was used as the 3′ splice site, resulted in the deletion of exon 51 and partial of 3′ UTR (**Figure S1B**).

### Intron Retention Caused by Alternative Splice Site in Intron

#### Recognition of Alternative Donor Site in Intron

In proband PUMC-401 (c.642+4delA in *COL1A1*), one mutant transcript with alternative donor site in intron 7 was recognized (**Figure 5Ac**). The alternative splice site c.589-62_589-61gt, which is located in intron 7, was selected preferentially as donor site during splicing. As a result, part of intron 7 (96bp) was inserted in the mutant transcript.

#### Recognition of Alternative Acceptor Site in Intron

**Figure 6:**
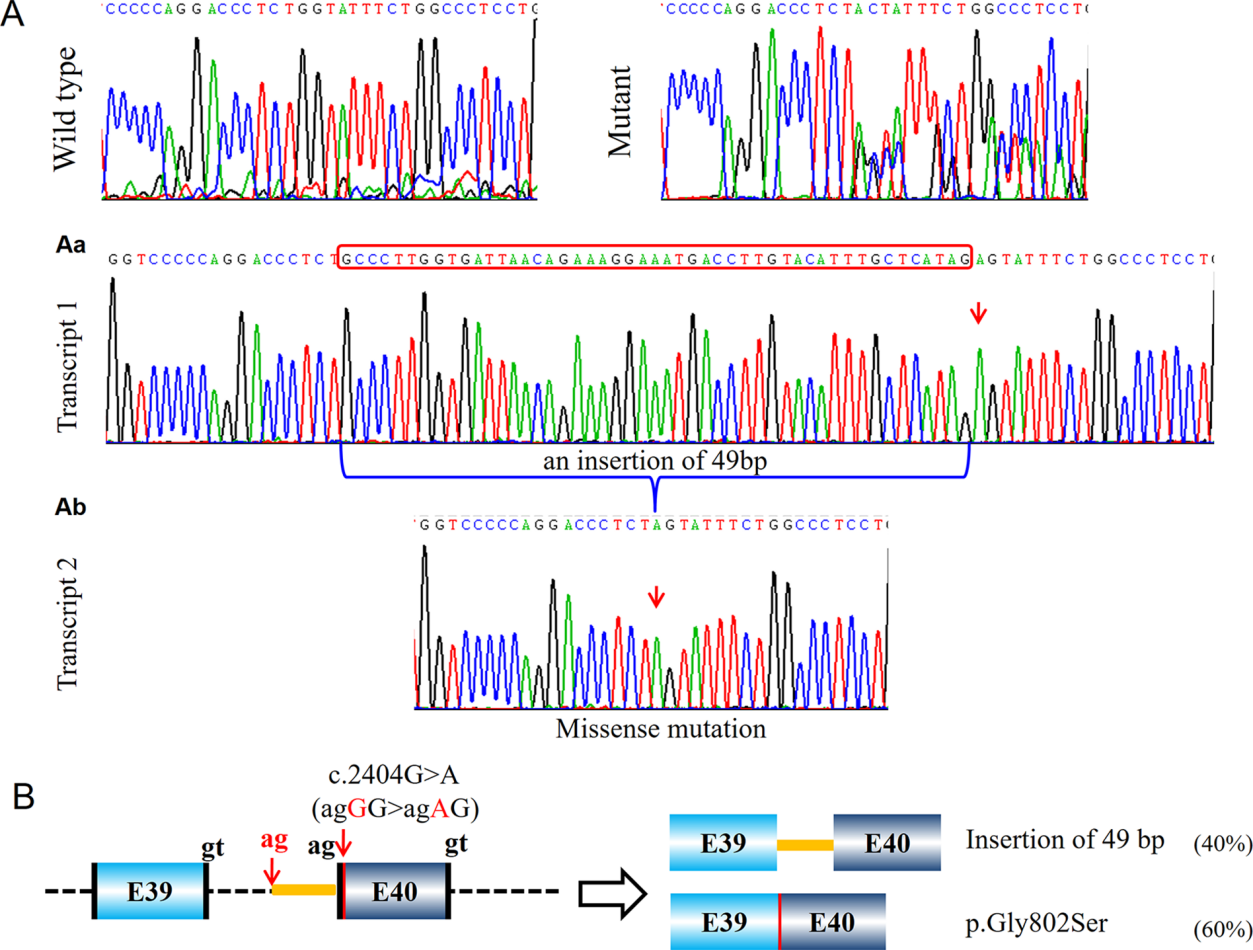
Identification of a compound aberrant splicing with a missense transcript and a transcript with intron retention (PUMC-296). **(A)** Minigene analysis showed a wild type transcript (first panel) and a mutant transcript (second panel). Because the mutant type had no specific signal from the mutant nucleotide, T-vector was used to identify the different transcripts. A missense transcript was found by T-vector cloning **(Ab)**, and an insertion of 49 nucleotides was found as the other transcript **(Aa)**. **(B)** Schematic representation of the splicing effect, indicating the missense mutation c.2404G > A in *COL1A2* resulted in a missense transcript and an intron retention transcript. The canonical splicing donor gt and splicing acceptor ag were labeled in black, and the newly activated cryptic donor site in red.

Five probands (PUMC-15, PUMC-105, PUMC-369, PUMC-189, and PUMC-296) were found with intron retention caused by alternative acceptor site in intron in this study (**Table 1**). In particular, PUMC-296 (**Figure 6**) carried a missense mutation c.2404G > A in *COL1A2* indicated by Sanger sequencing. Such change took place in the first nucleotide in exon 40, therefore agGG altered to agAG. An alternative 3′ splice site in intron 39, c.2404-51_2404-50ag, was recognized during splicing in one of the mutant transcripts (**Figure 6Aa**). This led to an insertion of 49bp (retention of partial intron 39) in the mRNA.

### Compound Splicing Effects Resulted From Numbers of Aberrant Splicing Transcripts

During splicing, more than one transcript can be generated because of the existence of alternative splicing. This makes some aberrant splicing cases even more complicated. In this study, there were three variants generating more than one mutant transcript showed by minigene assay: c.642+4delA in *COL1A1* (PUMC-401), c.3036_3045+2del in *COL1A1* (PUMC-480), and c.2404G > A in *COL1A2* (PUMC-296) (**Table 1**).

Patient PUMC-401 with a variant c.642+4delA in *COL1A1* formed four mutant splicing isoforms (**Figures 5Aa–Ad**). Four pairs of alternative splice sites utilized in this patient were labelled in the schematic map (**Figure 5B**): Splicing of gt^1^ (c.588+1_588+2gt) and ag^1^ (c.643-2_643-1ag) generated transcript 1 with skipping of exon 8 (**Figure 5Aa**); Splicing of gt^2^ (c.588+1_588+2gt) and ag^2^ (c.660_661AG) formed transcript 2 with deletion of exon 8 and partial exon 9 (**Figure 5Ab**); Splicing of gt^3^ (c.589-62_589-61gt) and ag^3^ (c.643-2_643-1ag) generated transcript 3 with deletion of exon 8 and insertion of partial intron 7 (**Figure 5Ac**); Transcript 4 was generated by splicing of gt^4^ (c.588+1_588+2gt) and ag^4^ (c.589-2_589-1ag), together with gt^4^ (c.617_618GT) and ag^4^ (c.643-2_643-1ag) with the effect of truncated exon 8 (**Figure 5Ad**). Exon skipping (transcript 1, 55%) and intron retention (transcript 3, 27%) were the most dominant isoforms (**Figure 5**). Sanger sequencing results of dermal fibroblasts confirmed that multiple transcripts were generated (**Figure 5C**), and that alternative donor/acceptor sites were utilized *in vivo* (**Figure S2**). Similarly, we found exon skipping (transcript 1, 17%) and intron retention (transcript 2, 25%) were the most prevalent mutant isoforms (**Figure S2**). However, 36 bp deletion in exon 8 (transcript 4, 4%) and retention of intron 8 (transcript 5, 4%) were only found in fibroblast assay (**Figure S2**) and skipping of exon 8 and partial exon 9 (transcript 2, 9%) were only found in minigene assay (**Figure 5**).

Similarly, patient PUMC-480 formed two different mutant transcripts (**Figure 4A**). Two pairs of alternative splice sites were used: Utilizing gt^1^ (c.2937+1_2937+2gt) and ag^1^ (c.3046-2_3046-1ag) generated transcript 1 with exon 41 skipping (33%); Utilizing gt^2^ (c.3029-3030 GT) and ag^2^ (c.3046-2_3046-1ag) generated transcript 2 with deletion of partial exon 41 (67%). Another patient, PUMC-296 (c.2404G > A in *COL1A2*) showed a missense variant at the first nucleotide in exon 40. Two transcripts were found by minigene analysis: one with a missense variant (c.2404G > A, 60%) and the other with an insertion of 49 bp (40%) by recognition of alternative 3′ splice site (c.2404-51_2404-50ag) in intron 39 (**Figure 6A**).

### No Remarkable Aberrant Splicing Effect

Among the 29 variants, 12 variants did not show any splicing consequence indicated by minigene assay (**Table 1**). Most of them were missense variants at the first nucleotide in the exons. While some of them (c.370-9C > T in *COL1A1*, c.2613+9C > T in *COL1A1*, c.1036-9G > T in *COL1A2*, c.2026-1_2042dup in *COL1A2*) carried the variants in introns without aberrant splicing, and they were excluded from the pathogenic variants. In particular, variant c.2026-1_2042dup in *COL1A2* (PUMC-253) should be highlighted. This variant may cause aberrant splicing because the duplication covered the 3′ boundary of intron 33 to the 5′ partial exon 34 (**Figure S3**). After verification using minigene assay, two transcripts were observed: a wild type transcript from the normal allele and a mutant transcript from the mutant allele (**Figure S3A**). Because the mutant transcript showed the same pattern c.2026-1_2042dup as the sequencing results, no splicing effect was found. RT-PCR of RNA extracted from fibroblasts of this patient confirmed that no aberration was observed (**Figure S3C**).

## Relationship Between Genotypes and Phenotypes

According to the clinical features of OI including fracture frequency, presence of blue sclerae and bone deformity, the 29 variants (34 OI patients) were classified into different phenotypical groups (**Table 1**): 8 variants were grouped as type I, 8 were type III, and the remaining 13 were type IV OI. Most of the variants with aberrant splicing corresponded to a mild phenotype (e.g. type I or type IV OI). For example, PUMC-296 who was identified the variant c.2404G > A in *COL1A2* leading to multiple mutant transcripts, presented a mild phenotype: 0.3 fracture times per year without other skeletal problems.

Those exhibited severe phenotype (type III OI), the minigene analysis showed no aberrant splicing (confirmed as no aberration for intronic variants or missense mutation for exonic variants) or exon skipping effect. For instance, PUMC-371 (c.792G > A in *COL1A2* consequent to skipping of exon 16) displayed rather severe phenotypes: with more than 30 times total fracture times (2.9 times yearly of fracture frequency), short stature (Z score = −6.32), presence of dentinogenesis imperfecta and disability of walking.

Moreover, the patients with aberrant splicing effects caused by intronic variants (n = 17) often expressed relatively milder phenotypes: only 11.76% (2 in 17) of them were OI type III, and 88.24% (15 in 17) were OI type I or type IV. Regarding the exonic variants, a large proportion led to a severe type III phenotype, being 30.77% (4 in 13).

## Discussion

The splicing effects of 29 suspected atypical splicing variants associate with OI were examined in current study. Among 29 variants, 17 were identified with aberrant splicing, and 12 were not observed any abnormal splicing effect. The splicing effects can be classified as (i) exon skipping or (ii) alternative splice site induced intron retention or partial exon deletion. We further conducted skin fibroblast RT-PCR sequencing and confirmed the findings in the minigene assay, suggesting it is a reliable approach to assess the splicing consequences.

## The Mechanism of Aberrant Splicing Generation

Pre-mRNA splicing occurs when exons and introns are precisely recognized. Two theories were proposed about the splicing initiation: the intron definition and exon definition (Keren et al., 2010). In intron definition, 5′ splice site (GT) and 3′ splice site (AG) as well as branch site (YNYURAY) are recognized and mRNA splicing mechanism places across the introns. Variants locate at any of these sites will impair the transcription (Vijayraghavan et al., 1986). While in the exon definition, exons are identified by their naturally high GC proportion. Though exon definition was believed to be the main mechanism of the evolution of alternative splicing (Ram and Ast, 2007), the core intronic splicing signal was still widely studied and believed to be crucial for aberrant splicing. In this study, 89% (17/19) aberrant splicing was caused by the intronic variants (**Table 1**), supporting this notion. To explore the mechanisms underlying aberrant splicing, we further analyzed our results and found the following three main causative reasons for aberrant splicing.

### Canonical 5′ Splice Site Cannot Be Recognized

This can be resulted from the alteration of an adjacent nucleotide, for example in patient PUMC-371 (c.792G > A in *COL1A2*), such variant changed the consensus sequence AAGgt to AAAgt (**Figure 3**). It was known that the conservation of last nucleotide at 3′ exon is G > A/T (Roca et al., 2012; Roca et al., 2013). The alteration from G to A changed the conservation, and disrupted the base-pairing between U1 small nuclear RNA (snRNA) and the donor site (Roca et al., 2013). In addition, unrecognition of authentic donor can be also caused by the inexistence of 5′ splice site resulted from a deletion (**Figure 4**). PUMC-480 (c.3036_3045+2del in *COL1A1*) belongs to this instance, and the disappearance of the canonical donor site induced exon skipping or the activation of a cryptic donor site.

### Both Canonical 5′ and 3′ Splice Sites Are Deactivated

The deactivation of both splice sites can lead to rather complicated case, for instance, PUMC-401 (c.642+4delA in *COL1A1*). The deletion changed 5′ intronic consensus sequence gtaag to gtag (**Figure 5**). The conservation of +4 site in intron is A > T/G (Roca et al., 2012), so the variant led to deactivation of canonical donor site and the selection of alternative donor/acceptor sites for all mutant transcripts both from minigene results (**Figures 5Aa–Ad**) and from cultured fibroblasts (**Figure S2**). The alteration near 5′ intronic site caused the deactivation of acceptor site in adjacent intron (**Figure 5Ab**), but the reasons remain to be elucidated. Similar effects were reported by Schwarze et al. (1999) that variant c.642+1G > A in *COL1A1* led to multiple mutant transcripts caused by employing alternative donor sites. As both variants are located in intron 8 of *COL1A1*, and it was showed that introns 5, 6, and 9 were removed before introns 7 and 8 (Schwarze et al. 1999). This could be one of the reasons that both studies found the compound transcripts when variants are located in intron 8 of *COL1A1*.

### Canonical 3′ Splice Site Cannot Be Recognized

A 3′ splice site includes a branch point, a polypyrimidine tract and a splicing acceptor site (Wahl et al., 2009). One possible reason leading to the unrecognition of 3′ splice site is the changing of nucleotide adjacent to the splicing acceptor as happened in PUMC-296 (c.2404G > A in *COL1A2*) (**Figure 6**). The acceptor site is recognized through non-Watson-Crick interaction by pairing with donor site and branch point (Wilkinson et al., 2017). Wilkinson et al. (2017) reported that the first 10 nucleotides of 5′ exon are always well ordered to facilitate the mRNA processing. The boundary of an 3′ splice site and 5′ exon is always consensus as Y_10_NCAG/G, where Y stands for pyrimidine and N equals A/G/C/T (Sun and Chasin, 2000). Therefore, the alteration at the first nucleotide in PUMC-296 resulted in deactivation of canonical acceptor, and instead a cryptic acceptor site was selected. Another reason is that the variants may be located at polypyrimidine tract (PPT) region. Variants in probands PUMC-276, PUMC-290, PUMC-15, PUMC-105, PUMC-369, and PUMC-189 belong to this case. It was known that by binding to different locations of sequences, polypyrimidine tract-binding protein1 (PTBP1) can induce either exon skipping or inclusion (Hamid and Makeyev, 2017). Sanz et al. (2010) reported that variants affecting PPT region resulted in the exon skipping. Consistently, in PUMC-276 and PUMC-290, both of the two variants caused skipping of exon 51 and deletion of partial 5′UTR (**Figure S1**). The remaining four variants mentioned above led to insertion of part of PPT region (**Table 1**).

## Relationship Between Aberrant Splicing and Phenotype

Most of the aberrant splicing found in this study corresponds to mild phenotypes (Type I or type IV OI) (**Table 1**). Type I collagen is a protein of triple helix structure comprised of two alpha1 chains and one alpha2 chain (Marini et al., 2017). Its synthesis involves the correct post-translational modifications, folding and secretion (Ishikawa and Bächinger, 2013). Variants within its encoding genes, *COL1A1* and *COL1A2*, have two main types of collagen defects: quantitative defect and structure defect (Marini et al., 2007). The structure alterations generally cause more severe phenotypes due to excessive post translational modification (Ishikawa and Bächinger, 2013). The collagen defect mechanism can be classified into two types: (I) Synthesizing of single *COL1A1* allele consequences in haploinsufficiency. This involves nonsense-mediated mRNA decay, or frameshift/splicing mutation-induced pre-termination codon, and most of them being mild OI type (Rauch et al., 2010); (II) The helical mutations of *COL1A1* or *COL1A2* induced structural change of type I collagen. Missense mutations in triple-helical domain can result in dominant negative effect, thus impair the collagen folding and synthesis. The helical mutations are mostly glycine substitutions and the severity varies from mild to severe levels (Rauch et al., 2010; Lindahl et al., 2015).

Among the aberrant splicing in this research, we noticed that all OI patients with more than one mutant transcripts (e.g. PUMC-401, PUMC-480, and PUMC-296) have mild phenotypes, being either type I or type IV OI (**Table 1**). Haploinsufficiency could be the main reason, as one wild type allele may fulfill the normal functions. Regarding the mutant allele, although there were many different mutant transcripts, some of them led to premature termination codon (e.g. PUCM-296, **Figure 6**), and induced the degradation of those transcripts (Kervestin and Jacobson, 2012). Therefore, in principle, only a small proportion of defective transcripts affect the collagen function.

Similarly, variants locate at the polypyrimidine tract (PPT) region (e.g. PUMC-15, PUMC-105, PUMC-369, PUMC-189, PUMC-276 and PUMC-290) have mild phenotypes as well. What need to be noted here is PUMC-15, 105, 369, and 189, among whom all the variants resulted in an insertion of part of PPT sequence, generated the premature termination codon and therefore resulted in the degradation of the defective transcript (Kervestin and Jacobson, 2012). Their phenotypes (type I OI) are in agreement with the protein alteration (**Table 1**).

The most dominant splicing effect is exon skipping. However, we did not observe a strong correlation between exon skipping and phenotype (**Table 1**). Most of patients with exon skipping expressed milder clinical manifestations (type I or type IV OI) than those with missense mutations. There are only three (PUMC-90, 312, and 371) patients have severer phenotype, with two exon 16, and one exon 44 skipping, respectively. Depending on the location of skipped exons, the severity of OI can vary from mild to severe level (Thomas and DiMeglio, 2016). Even if the skipping did not change the Gly-X-Y triplet pattern, the chain alignment may still have causative effect on collagen folding (Marini et al., 2017). If the variant occurs at the C-terminal region of propeptide, this may be associated with protein folding delay, thus further affect the correct assembly of collagen (Symoens et al., 2014). The locations of both alteration (Marini et al., 2007) and modifier genes (Riordan and Nadeau, 2017) contribute to different phenotypes, and details remain to be elucidated.

Although a large proportion of structural defects of collagen was due to the classical splicing mutations (Marini et al., 2007), atypical variants in the introns or exons that are close to the splice sites are also important and hence should be highlighted in future sequencing analysis. Among the recruited 867 OI patients, we found 17 atypical splicing variants and 22 typical splicing variants. Thus the atypical splicing variants represent a high proportion (44%, 17/39). For the first time, our study examined and classified the atypical (exon/intron border exclusive) splicing variants associated with OI, which helps to identify the causative mutation and establish the correlation between splicing effect and OI phenotypes.

## Data Availability Statement

The Datasets Generated for This Study Can Be Found in the Osteogenesis Imperfecta Variant Database (Http://Oi.Gene.Le.Ac.Uk/). 

## Ethics Statement

All procedures performed in this study involving human participants were approved by Institutional Review Board (IRB) of the Institute of Basic Medical Sciences, Chinese Academy of Medical Sciences, Beijing, China (015-2015). Informed consent was obtained from all adult participants/legal guardians of children under age 18.

## Author Contributions

LL and YC performed the minigene assay, sequencing analysis, and wrote the manuscript. FZ, BM, and YY carried out plasmid construction. SL and TY conducted data collection as well as data analysis. XR and YW helped with recruiting patients and YG helped to discuss the data and helped writing the final manuscript. XZ conceived the study and supervised this research. All authors performed critical reading and approved the final version of manuscript. 

## Funding

This study was supported by grants from National Key Research and Development Program of China (2016YFE0128400, 2016YFC0905100), CAMS Innovation Fund for Medical Sciences (CIFMS, 2016-I2M-3-003) and National Natural Science Foundation of China (81472053).

## Conflict of Interest

The authors declare that the research was conducted in the absence of any commercial or financial relationships that could be construed as a potential conflict of interest.
