# Supplementary material for: Validation and Classification of Atypical Splicing Variants Associated With Osteogenesis Imperfecta
Source: Front Genet. 2019 Oct 18;10:979. doi: 10.3389/fgene.2019.00979 (PMC6832110; doi:10.3389/fgene.2019.00979)
Supplement: Supplementary file 1 [file DataSheet_1.docx]

**Supplementary Table 1. Primers used in this study.**

| **Proband number** | **Primer name** | **Sequence (5' → 3')** | **T_m_ (°C)** | **Size (bp)** |
| --- | --- | --- | --- | --- |
| *PUMC-229* | *F* | CCCAAGCTTCAAGTGTGCCTCTTAGACCC | 60 | 1716 |
|  | *R* | GGAATTCTGAGGTCCCAAAGGTGATCT |  |  |
| *PUMC-15* | *F* | CCCAAGCTTTCCCAAGGCTCTTTCTCAGA | 60 | 865 |
|  | *R* | GGAATTCATGCTTAGAGGAGAGTGGGG |  |  |
| *PUMC-105* | *F* | CCCAAGCTTTCCCAAGGCTCTTTCTCAGA | 60 | 865 |
|  | *R* | GGAATTCATGCTTAGAGGAGAGTGGGG |  |  |
| *PUMC-369* | *F* | CGGGATCCCCTTTCCCTCTGCTCCTAGG | 60 | 1099 |
|  | *R* | CGACGCGTCCTCATCCCAGACCCTACAC |  |  |
| *PUMC-189* | *F* | CGGGATCCCCTTTCCCTCTGCTCCTAGG | 60 | 1099 |
|  | *R* | CGACGCGTCCTCATCCCAGACCCTACAC |  |  |
| *PUMC-401* | *F* | CGGGATCCTTCTCAGCACATCACACCAG | 60 | 1503 |
|  | *R* | CGACGCGTTGGACCCAGCTCCTAAATGA |  |  |
| *PUMC-186* | *F* | CGGGATCCTGGTCTGAACCCCAGGACTT | 60 | 951 |
|  | *R* | CGACGCGTAGAGGATGAGCTGAGAGTCG |  |  |
| *PUMC-109* | *F* | CGGGATCCCCTTCCCTCCCTTCACACAG | 60 | 1069 |
|  | *R* | CGACGCGTACCAACTTCACCAGGACGTC |  |  |
| *PUMC-469* | *F* | CGGGATCCCCTTCCCTCCCTTCACACAG | 60 | 1069 |
|  | *R* | CGACGCGTACCAACTTCACCAGGACGTC |  |  |
| *PUMC-111* | *F* | CGGGATCCGAAACCCAGACACAAGCAGA | 60 | 1107 |
|  | *R* | CGACGCGTGAGCACCAACATTACCCTGT |  |  |
| *PUMC-480* | *F* | CGGGATCCGCTGATGGTCCTGCTGTAAG | 60 | 991 |
|  | *R* | CGACGCGTTGACATCTTGCAGGATCTCC |  |  |
| *PUMC-276* | *F* | CGGGATCCCTCCCTTTCCCTGACTCCAT | 60 | 2510 |
|  | *R* | TTGCGGCCGCCTTCCCCATGTCTACCCCTC |  |  |
| *PUMC-290* | *F* | CGGGATCCCTCCCTTTCCCTGACTCCAT | 60 | 2510 |
|  | *R* | TTGCGGCCGCCTTCCCCATGTCTACCCCTC |  |  |
| *PUMC-391* | *F* | CGGGATCCTGCAGCAGACAAGACTTACC | 60 | 2183 |
|  | *R* | CGACGCGTGGGAAGATCAGGTGGAAACT |  |  |
| *PUMC-90* | *F* | CGGGATCCTCTGCTTCTAGGGAGCCCGT | 60 | 1447 |
|  | *R* | CGACGCGTGTATACTCACAGCAGCACCC |  |  |
| *PUMC-224* | *F* | CGGGATCCTAATGTGTGCTGCCTCTACA | 60 | 903 |
|  | *R* | CGACGCGTGTGTGTTAGGGTGTTGGGAG |  |  |
| *PUMC-98* | *F* | CGGGATCCTAATGTGTGCTGCCTCTACA | 60 | 903 |
|  | *R* | CGACGCGTGTGTGTTAGGGTGTTGGGAG |  |  |
| *PUMC-216* | *F* | CGGGATCCTCATCCTGAAATACCACCTC | 60 | 1153 |
|  | *R* | CGACGCGTACACCCACCACCAGAAGCAG |  |  |
| *PUMC-37* | *F* | CGGGATCCTCATCCTGAAATACCACCTC | 60 | 1153 |
|  | *R* | CGACGCGTACACCCACCACCAGAAGCAG |  |  |
| *PUMC-253* | *F* | CGGGATCCGTAAGGAATCGAGACATTGC | 60 | 2099 |
|  | *R* | CGACGCGTTGATACTCACAGCAGGACCA |  |  |
| *PUMC-312* | *F* | CGGGATCCTGAAAGAGGGTTCGTTACTG | 60 | 1068 |
|  | *R* | CGACGCGTTTTATGTGCGAGATGGCTAC |  |  |
| *PUMC-2* | *F* | CGGGATCCGGGTGGCTTCTGATATGTCC | 60 | 1065 |
|  | *R* | CGACGCGTGAGAGGAGGCCAGTGAACTC |  |  |
| *PUMC-234* | *F* | CGGGATCCAGTGGACTTAACGGGGCTTC | 60 | 925 |
|  | *R* | CGACGCGTACCAGCAATACCAGGAGCAC |  |  |
| *PUMC-351* | *F* | CGGGATCCCCTTCCCTCCCTTCACACAG | 60 | 1069 |
|  | *R* | CGACGCGTACCAACTTCACCAGGACGTC |  |  |
| *PUMC-23* | *F* | CGGGATCCCCTTCCCTCCCTTCACACAG | 60 | 1069 |
|  | *R* | CGACGCGTACCAACTTCACCAGGACGTC |  |  |
| *PUMC-41* | *F* | CGGGATCCCCTTCCCTCCCTTCACACAG | 60 | 1069 |
|  | *R* | CGACGCGTACCAACTTCACCAGGACGTC |  |  |
| *PUMC-339* | *F* | CGGGATCCCCTTCCCTCCCTTCACACAG | 60 | 1069 |
|  | *R* | CGACGCGTACCAACTTCACCAGGACGTC |  |  |
| *PUMC-371* | *F* | CGGGATCCCTGGTGAGAGAGGACGTGTT | 60 | 1378 |
|  | *R* | CGACGCGTCTCCAGGATTACCCTATGAG |  |  |
| *PUMC-479* | *F* | CGGGATCCGACAGAAGGAGAGGGAAGGT | 60 | 903 |
|  | *R* | CGACGCGTAGGAAGGGCATGTCTGTGTG |  |  |
| *PUMC-115* | *F* | CGGGATCCTGGTGAAGTGAGTGCCATTT | 60 | 1039 |
|  | *R* | CGACGCGTTGGGGCTAACTTTAATGGGT |  |  |
| *PUMC-296* | *F* | CGGGATCCTGCCTACCTCCTACTCCTTG | 60 | 2691 |
|  | *R* | CGACGCGTTACTGTCAAGCACTCACCAC |  |  |
| *PUMC-448* | *F* | CGGGATCCGGCAGTATTTGGGCTTTCGT | 60 | 808 |
|  | *R* | CGACGCGTAGTGGGGTATTAAACAGGGG |  |  |
| *PUMC-441* | *F* | CGGGATCCCTGGTCCTGCTGTGAGTATC | 60 | 1579 |
|  | *R* | CGACGCGTATCAGAAGCCAGGCCTTTTG |  |  |
| *PUMC-430* | *F* | CGGGATCCGCCTCACCAACAGCCTTAAT | 60 | 986 |
|  | *R* | CGACGCGTGTGCGAGATGGCTACAGTTT |  |  |

**Supplementary Table 2**

**Information of the typical splicing variants in *COL1A1* and *COL1A2* in this study**

| **Gene** | **Variant type** | **Variant position** | **Nucleotide change** | **Family number** | **Total number of patients** | **OI type** |
| --- | --- | --- | --- | --- | --- | --- |
| *COL1A1* | splicing | intron 1 | c.104-1G>A | PUMC-212 | 2 | IV |
|  | splicing | intron 1 | c.103+2T>G | PUMC-372 | 2 | I |
|  | splicing | intron 10 | c.750+1G>A | PUMC-96,358 | 4 | III, IV |
|  | splicing | intron 17 | c.1155+1G>A | PUMC-169,292 | 8 | I, I |
|  | splicing | intron 18 | c.1200+1G>T | PUMC-26 | 4 | I |
|  | splicing | intron 19 | c.1300-1G>A | PUMC-87 | 4 | I |
|  | splicing | intron 19 | c.1299+1G>A | PUMC-228 | 4 | I |
|  | splicing | intron 19 | c.1299+1G>A | PUMC-472 | 2 | I |
|  | splicing | intron 21 | c.1461+1G>A | PUMC-61 | 3 | I |
|  | splicing | intron 23 | c.1614+1G>A | PUMC-453 | 3 | I |
|  | splicing | intron 26 | c.1821+1G>A | PUMC-149 | 5 | I |
|  | splicing | intron 3 | c.333+1G>A | PUMC-126 | 2 | IV |
|  | splicing | intron 39 | c.2829+1G>T | PUMC-99 | 2 | I |
|  | splicing | intron 40 | c.2938-1G>C | PUMC-484 | 3 | III |
|  | splicing | intron 47 | c.3531+1G>T | PUMC-83 | 3 | I |
|  | splicing | intron 48 | c.3815-2A>G | PUMC-127 | 2 | I |
| *COL1A2* | splicing | intron 11 | c.541-2delA | PUMC-168 | 5 | I |
|  | splicing | intron 14 | c.694-2A>C | PUMC-483 | 1 | IV |
|  | splicing | intron 15 | c.739-2A>G | PUMC-47 | 2 | III |
|  | splicing | intron 16 | c.792+2T>C | PUMC-119 | 2 | IV |
|  | splicing | intron 40 | c.2565+1G>A | PUMC-424 | 1 | III |
|  | splicing | intron 43 | c.2835+1G>A | PUMC-100,135,332 | 8 | IV, IV, IV |

**Supplementary Table 3**

**Splicing effect predicted by in silico tools**

| **Proband number** | **Gene** | **Location** | **Mutation** | **Effect observed in Minigene assay** | **Human Splicing Finder*** | **ESE finder** | |
| --- | --- | --- | --- | --- | --- | --- | --- |
|  |  |  |  |  |  | **Matrix library/**  **Matrices/Threshold** | **Results#** |
| **Variants in introns** | | | |  |  |  |  |
| PUMC-229 | *COL1A1* | Intron 4 | c.370-9C>T | No aberration | a | SpliceSites/BranchSite/0 | 1.11820-1.11820 |
| PUMC-401 | *COL1A1* | Intron 8 | c.642+4delA | Retention of intron 7 and skipping of exon 8/skipping of exon 8 and partial exon 9/Skipping of exon 8/ Partial exon 8 del | h | SpliceSites/BranchSite/0 | 1.61280-0 |
| PUMC-15 | *COL1A1* | Intron 13 | c.904-10T>A | Retention of 8bp | c, i | NA | NA |
| PUMC-105 | *COL1A1* | Intron 13 | c.904-15T>A | Retention of 13bp | c, i | NA | NA |
| PUMC-186 | *COL1A1* | Intron 17 | c.1155+3delA | Skipping of exon 17 | h | SpliceSites/BranchSite/0 | 1.2745-0 |
| PUMC-369 | *COL1A1* | Intron 20 | c.1354-12G>A | Retention of 10bp | c, i | SpliceSites/BranchSite/0 | 0-4.1575 |
| PUMC-189 | *COL1A1* | Intron 20 | c.1354-12G>A | Retention of 10bp | c, i | SpliceSites/BranchSite/0 | 0-4.1575 |
| PUMC-111 | *COL1A1* | Intron 34-exon 35 | c.2398-2_2406del | Skipping of exon 35 | c, i | SRPprotein/SRSF2/2.383 | 2.77360-0 |
| PUMC-109 | *COL1A1* | Intron 37 | c.2613+6 T>C | Skipping of exon 37 | h | SpliceSites/BranchSite/0 | 2.28700-2.87690 |
| PUMC-469 | *COL1A1* | Intron 37 | c.2613+9C>T | No aberration | a | SpilceSites/5SS_U2_Human/6.67 | 8.31830-7.98510 |
| PUMC-480 | *COL1A1* | Exon 41-intron 41 | c.3036_3045+2del | Skipping of exon 41/partial exon 41 del | NA | SRPprotein/SRSF2/2.383 | 0-2.91086 |
| PUMC-276 | *COL1A1* | Intron 50 | c.4249-26_4249-8del | Exon 51_3’UTRdel | b | SpliceSites/BranchSite/0 | 1.70810-0 |
| PUMC-290 | *COL1A1* | Intron 50 | c.4249-3_4249-2del | Exon 51_3’UTRdel | i | SpliceSites/BranchSite/0 | 1.34850-1.07790 |
| PUMC-391 | *COL1A2* | Intron 13 | c.639+5_639+25del | Skipping of exon 13 | h | SpliceSites/BranchSite/0 | 3.99870-0 |
| PUMC-90 | *COL1A2* | Intron 16 | c.792+3A>T | Skipping of exon 16 | h | SpliceSites/BranchSite/0 | 0.28380-0.93660 |
| PUMC-224 | *COL1A2* | Intron 19 | c.1036-9G>T | No aberration | a | SpliceSites/BranchSite/0 | 2.12790-0.66170 |
| PUMC-98 | *COL1A2* | Intron 20 | c.1089+6T>G | Skipping of exon 20 | h | NA | NA |
| PUMC-216 | *COL1A2* | Intron 21 | c.1197+5G>A | Skipping of exon 21 | h | SpliceSites/BranchSite/0 | 2.03430-1.24850 |
| PUMC-37 | *COL1A2* | Intron 21 | c.1197+5G>A | Skipping of exon 21 | h | SpliceSites/BranchSite/0 | 2.03430-1.24850 |
| PUMC-253 | *COL1A2* | Intron 33–exon 34 | c.2026-1_2042dup | No aberration | f | SRPprotein/SRSF2/2.383 | 4.17117-4.17117 |
| PUMC-312 | *COL1A2* | Intron 44 | c.2943+1_2943+2delgt | Skipping of exon 44 | g, d | SpliceSites/BranchSite/0 | 1.27450-1.84440 |
| **Variants in exons** | | | |  |  |  |  |
| PUMC-234 | *COL1A1* | Exon 16 | c.1003G>A | No aberration | e | SRPprotein/SRSF1/1.956 | 5.25819-2.68045 |
| PUMC-2 | *COL1A1* | Exon 19 | c.1201G>A | No aberration | e, f | SRPprotein/SRSF1/1.956 | 5.25819-2.68045 |
| PUMC-351 | *COL1A1* | Exon 37 | c.2560G>A | No aberration | e | SRPprotein/SRSF5/2.67 | 5.65565-3.27392 |
| PUMC-23 | *COL1A1* | Exon 37 | c.2560G>A | No aberration | e | SRPprotein/SRSF5/2.67 | 5.65565-3.27392 |
| PUMC-41 | *COL1A1* | Exon 37 | c.2560G>A | No aberration | e | SRPprotein/SRSF5/2.67 | 5.65565-3.27392 |
| PUMC-339 | *COL1A1* | Exon 37 | c.2560G>A | No aberration | e | SRPprotein/SRSF5/2.67 | 5.65565-3.27392 |
| PUMC-371 | *COL1A2* | Exon 16 | c.792G>A | Skipping of exon 16 | h, f, g | SRPprotein/SRSF1(IgM-BRCA1)/1.867 | 2.52023-1.88465 |
| PUMC-479 | *COL1A2* | Exon 18 | c.892G>A | No aberration | e, f | SRPprotein/SRSF1/1.956 | 2.94818-0 |
| PUMC-448 | *COL1A2* | Exon 28 | c.1612G>A | No aberration | e, f | SRPprotein/SRSF1/1.956 | 0-2.89193 |
| PUMC-441 | *COL1A2* | Exon 38 | c.2296G>A | No aberration | e, f | SRPprotein/SRSF2/2.383 | 2.44024-0 |
| PUMC-296 | *COL1A2* | Exon 40 | c.2404G>A | Retention of 49bp/No aberration | e, f | SRPprotein/SRSF1/1.956 | 2.94818-0 |
| PUMC-430 | *COL1A2* | Exon 45 | c.2944G>A | No aberration | e | SRPprotein/SRSF1/1.956 | 5.25819-2.68045 |
| PUMC-115 | *COL1A2* | Exon 48 | c.3106G>T | No aberration | f | SRPprotein/SRSF1(IgM-BRCA1)/1.867 | 2.25749-0 |

*Note in Human Splicing Finder: **a**. No significant splicing motif alteration detected. This mutation has probably no impact on splicing. **b**. Creation of an intronic ESE site. Probably no impact on splicing. **c**. Activation of an intronic cryptic acceptor site. Potential alteration of splicing. **d**. Activation of an intronic cryptic donor site. Potential alteration of splicing. **e**. Activation of an exonic cryptic acceptor site. Potential alteration of splicing. **f**. Creation of an exonic ESS site. Potential alteration of splicing. **g**. Alteration of an exonic ESE site. Potential alteration of splicing. **h**. Alteration of the WT donor site, most probably affecting splicing. **i**. Alteration of the WT acceptor site, most probably affecting splicing. **NA**. Not Available.

# Values in results indicate the strength of natural site – strength of mutant site. NA. Not Available.


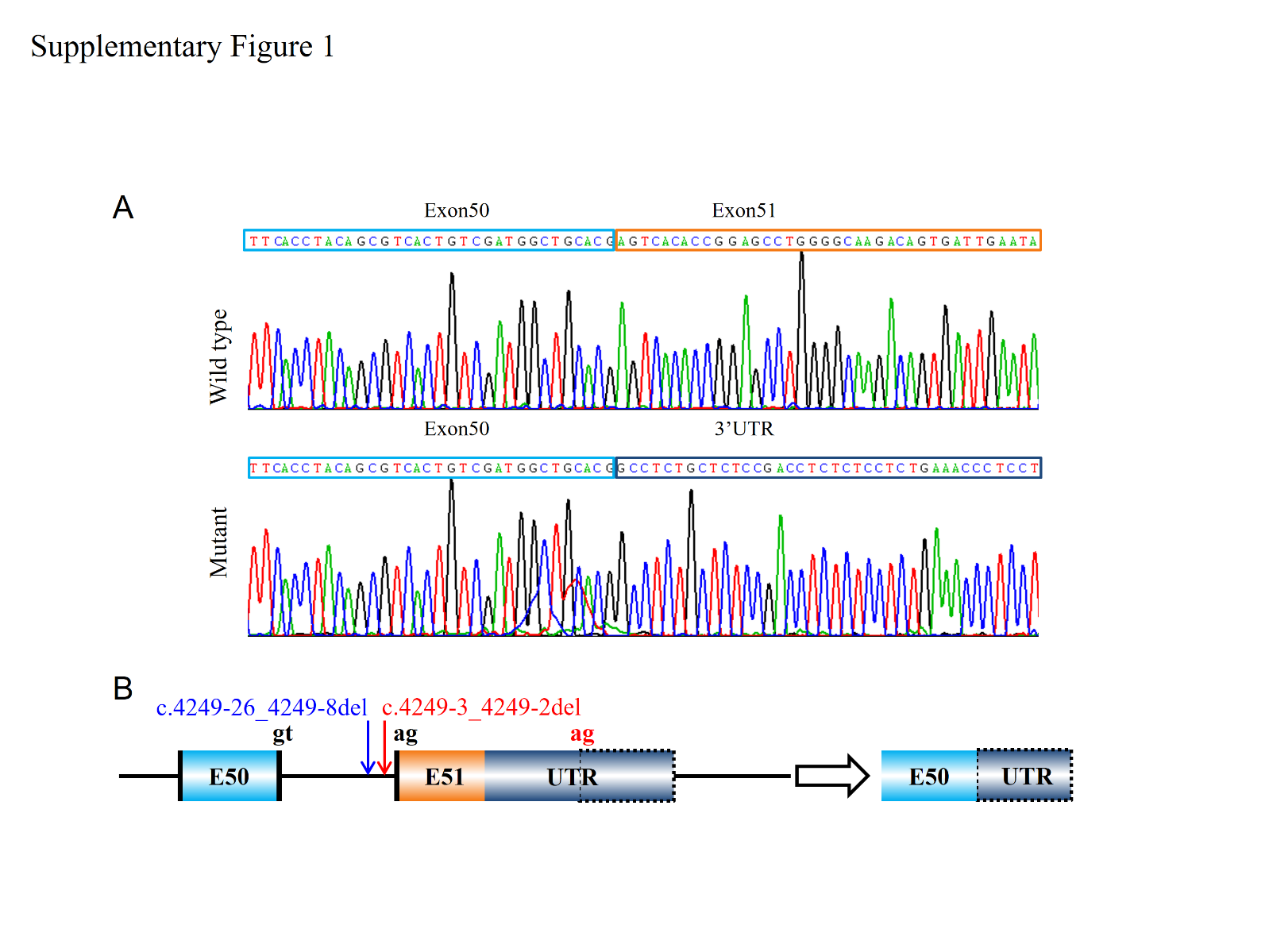


**Figure S1 Recognition of alternative splice site led to exon skipping and insertion of truncated UTR (PUMC-276, 290). (A)** Minigene analysis showed a wild type transcript (upper panel) and a mutant transcript (lower panel). (**B)** Schematic representation of the splicing effect of the two variants, c.4249-26_4249-8del, c.4249-3_4249-2del in *COL1A1*. Alternative splicing acceptor (c.4395+1147_4395+1148AG) in UTR region was recognized, resulted in exon 51 skipping and insertion of truncated UTR. The canonical splicing donor and splicing acceptor were labelled in black, and the newly activated splice acceptor in red.


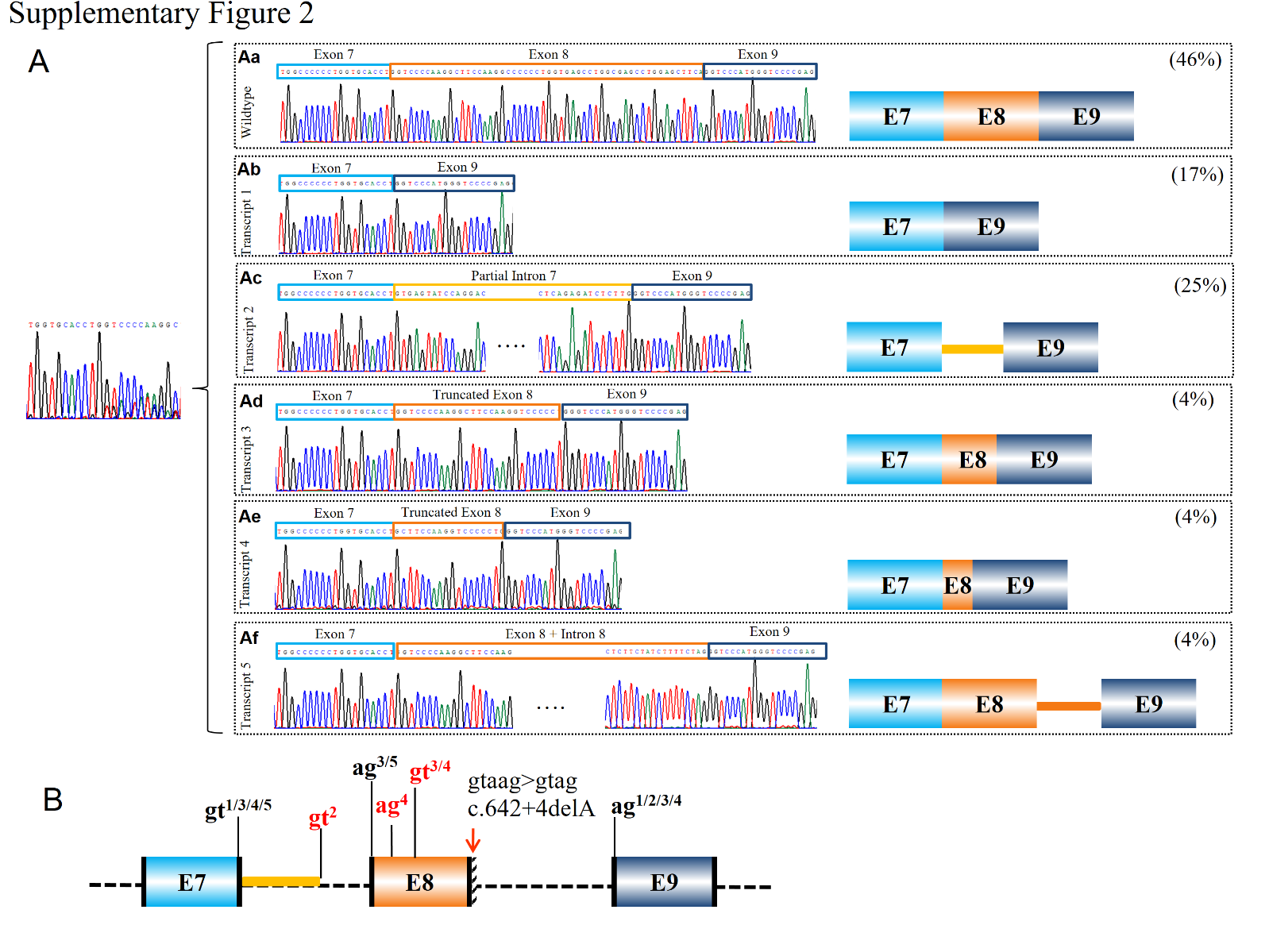


**Figure S2 Identification of a complex splicing effect with exon skipping, truncated exon and intron retention confirmed by dermal fibroblasts (PUMC-401). (A)** Sequencing analysis indicated that multiple transcripts can be generated: six different transcripts were further identified including the wildtype transcript (**Aa)** and five mutant transcripts **(Ab** -**Af**). (**B)** Schematic representation of the aberrant splicing effects. Both authentic splice sites and cryptic splice sites were marked on the representation: canonical splicing donor gt and splicing acceptor ag were labelled in black, and the newly activated cryptic donor site in red. Notations gt^n^ indicate the splicing donor sites utilized in transcripts n; ag^n^ indicates the splicing acceptor sites utilized in transcripts n (n=1-5).


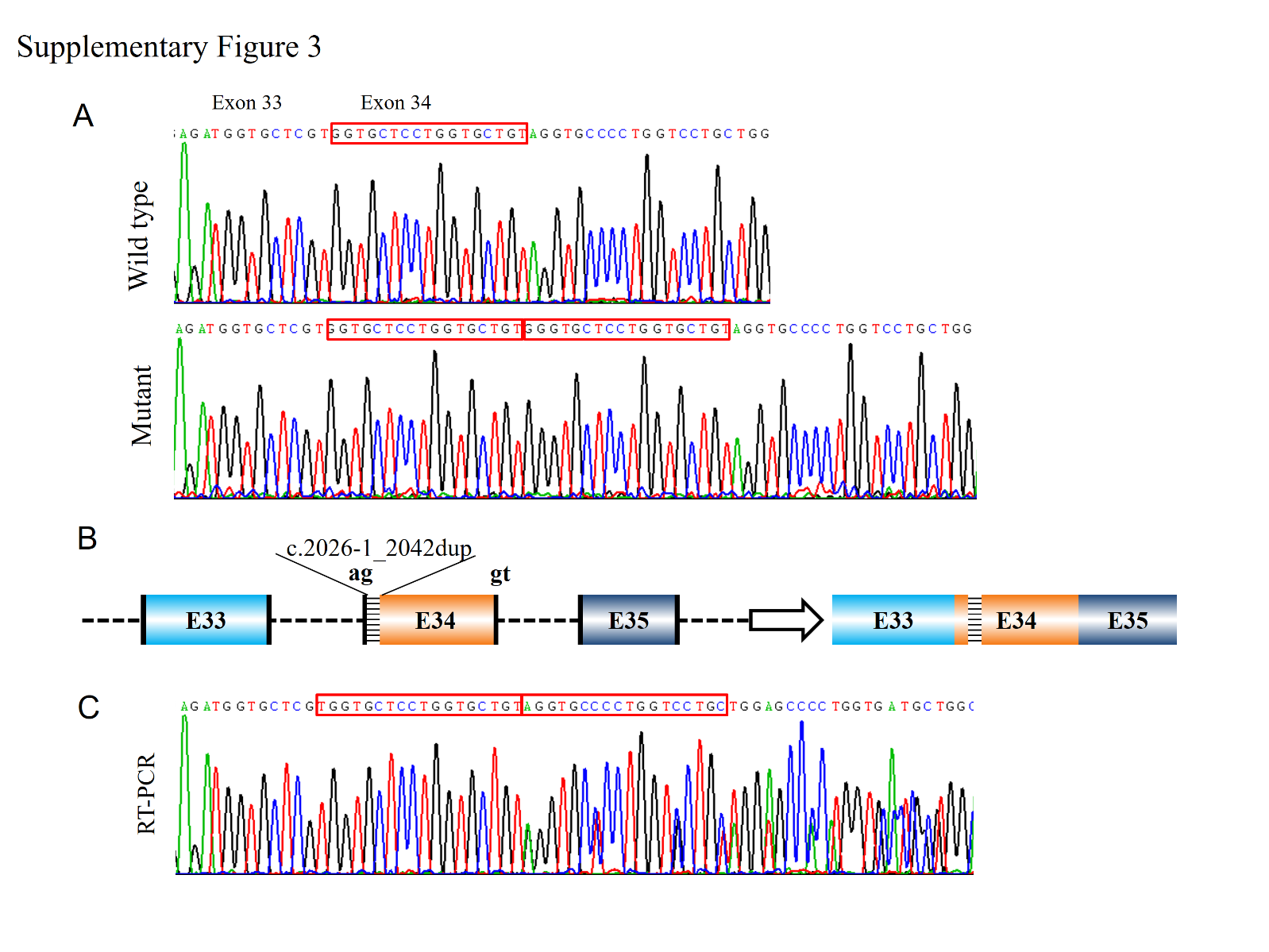


**Fig. S3 No aberrant splicing was found in a variant with duplication at exon-intron boundary (PUMC-253) A.** The minigene assay of the wildtype allele and the mutant allele: wildtype transcript (upper panel) and transcript of the mutant allele (c.2016-1_2042dup in *COL1A2* ) with a duplication of 18 nucleotides (lower panel). **B.** Schematic representation of the splicing effect, showing this duplication did not affect splicing. **C.** Sequencing analysis validated by RT-PCR from skin fibroblasts showed the same duplication in transcriptional level.
